# Clinical efficacy of intra-articular infusion of cocktail combined with tranexamic acid in the treatment of middle-age and older patients with frozen shoulder following arthroscopic capsular release

**DOI:** 10.3389/fsurg.2023.1035054

**Published:** 2023-05-03

**Authors:** Xiaojin Bai, Fan Bai, Zhi Wang, Yan Zhang, Fuying Liu, Qibing Wang

**Affiliations:** ^1^ Department of Joint Surgery, Third Affiliated Hospital of Zunyi Medical University (The First People’s Hospital of Zunyi), Zunyi, China; ^2^ Department of Neurology, Third Affiliated Hospital of Zunyi Medical University (The First People’s Hospital of Zunyi), Zunyi, China; ^3^ Authentication Center for Forensic Medicine and Judicature, Third Affiliated Hospital of Zunyi Medical University (The First People’s Hospital of Zunyi), Zunyi, China

**Keywords:** frozen shoulder, arthroscopy, cocktail, tranexamic acid, early postoperative

## Abstract

**Objective:**

To investigate the clinical efficacy of arthroscopic capsular release and postoperative intra-articular infusion of cocktail combined with tranexamic acid (TXA) in the treatment of patients with frozen shoulder.

**Method:**

A total of 85 middle-aged and older patients with frozen shoulder who underwent arthroscopic capsular release and received intra-articular infusion of TXA alone (*n* = 28), cocktail alone (*n* = 26), and cocktail plus TXA (*n* = 31) after surgery were retrospective analyzed. The drainage volume within 24 h after surgery, postoperative length of hospital stay, postoperative complications, visual analog scale (VAS), Neer shoulder assessment scale, ASES score, and range of motion (ROM) of the shoulder joint at 1 day, 1 week, 1 month, and 3 months after surgery in all three groups were recorded and compared.

**Results:**

Postoperative length of hospital stay was significantly shorter in the cocktail + TXA and cocktail groups than that in the TXA group. Postoperative drainage volume was significantly higher in the cocktail group compared with TXA + cocktail group (P < 0.05). At 1 day and 1 week after surgery, pain was more pronounced in the TXA group, which was significantly relieved in the cocktail and the cocktail + TXA groups (P < 0.05). Pain was significantly relieved in all the three groups at 1 and 3 months after surgery. Significant functional improvement of the shoulder was achieved in all three groups at 1 week after surgery, the improvement was apparent in the cocktail + TXA groups (P < 0.05), followed by the cocktail group. At 1 month after surgery, patients in the cocktail + TXA groups obtained excellent functional recovery of the shoulder joint. At 3 months after surgery, patients in all the three groups both obtained good recovery of the shoulder joint function, and the recovery was apparent in the cocktail + TXA groups (P < 0.05).

**Conclusion:**

Arthroscopic capsular release and postoperative intra-articular infusion of cocktail combined with TXA has good safety and efficacy in the treatment of middle-age and older patients with frozen shoulder, which can reduce postoperative pain and intra-articular bleeding, promote early postoperative functional exercises and accelerate early postoperative recovery.

## Introduction

Frozen shoulder is a common chronic injury disorder of the locomotor system in middle-aged and older people, which is also called adhesive capsulitis, painful stiff shoulder, and periarthritis. The main characteristic of frozen shoulder are limited full range of motion (ROM) in the shoulder joint, chronic pain around the insertion point of the deltoid muscle of the shoulder joint, which seriously affects the patients’ limb function, work ability and patients' quality of life (1). At present, the etiology and pathogenesis of frozen shoulder is not yet clear. Its pathological changes include thickening of capsule of the glenohumeral joint with partial contracture, and some patients may be presented with diffuse inflammation and fibrosis of the ligament tissue surrounding the joint capsule (2). 3D volumetric MR arthrographic assessment of shoulder joint capacity in patients with frozen shoulder showed that the rotator interval, the bicipital groove, and total glenohumeral joint volume were all reduced in patients with frozen shoulder compared with normal controls, extensive adhesion around the joints occurred, resulting in impaired movement of the shoulder joint (3).

**Figure 1:**
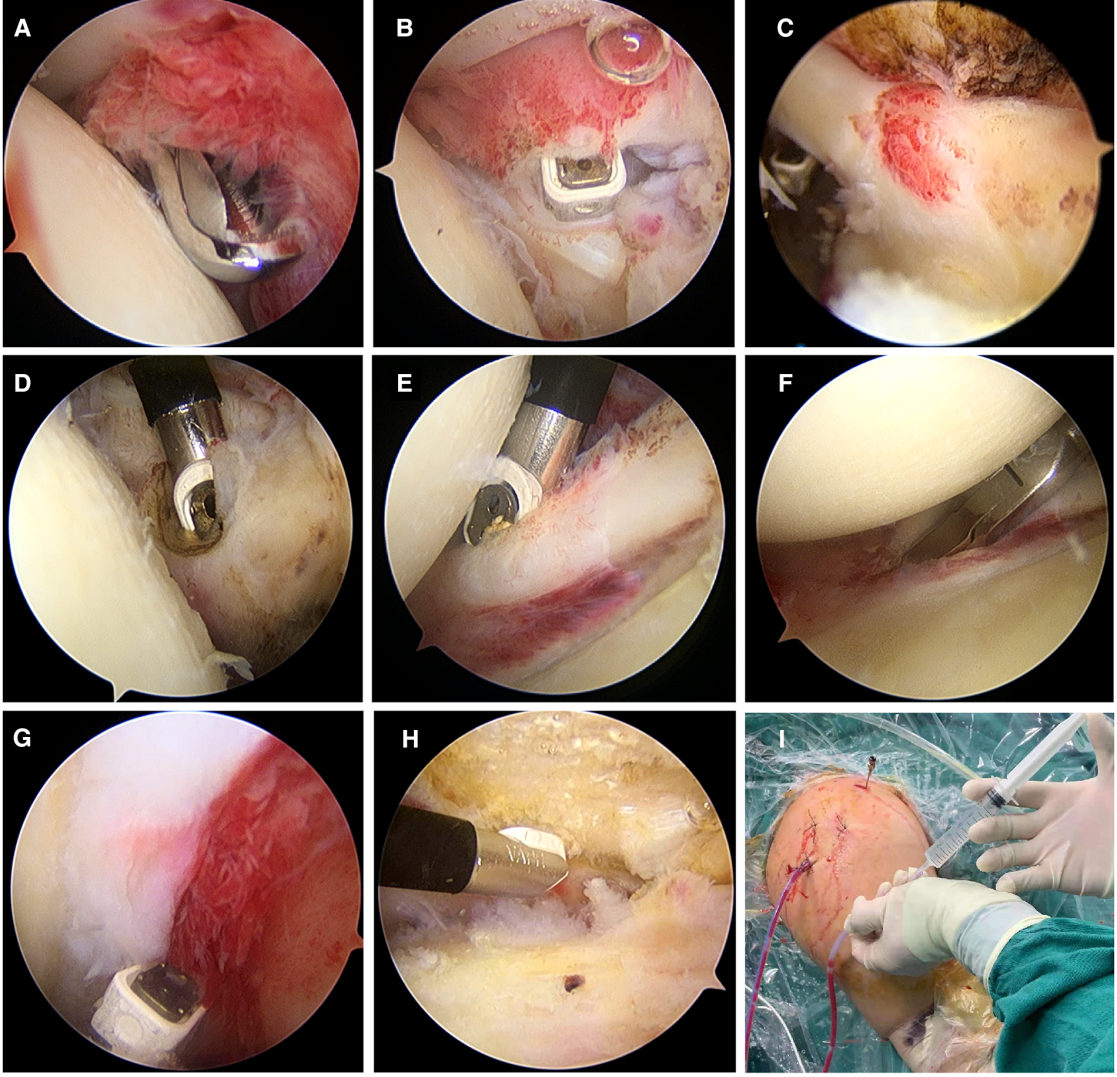
Surgical procedures of arthroscopic capsular release. (**A**) Clear the inflammatory synovium; (**B**) Release of the rotator interval; (**C**) Release of the long head of the biceps tendon; (**D**) Release of the middle glenohumeral ligament; (**E**) Release of the anterior band of the inferior glenohumeral ligament; (**F**) releasing of the anterior and inferior articular capsule; (**G**) Release of the posterior capsule; (**H**) Release of the subacromial space; (**I**) Intra-articular infusion of drugs after surgery.

Most patients with frozen shoulder can achieve good results by conservative treatments such as physical therapy, intra-articular injection and extracorporeal shock wave therapy. The failure of conservative treatment and the presence of severe symptoms and typical signs of frozen shoulder are all indications for surgical treatment. Severe symptoms include: (1) persistent pain (worsening during the night) and poor sleep quality; (2) joint contractures due to extensive soft-tissue thickening and adhesion; (3) limitation of active and passive movements of the shoulder joint in all directions, that seriously affects people's daily life, such as dressing, washing the face, and touching the lower back. Typical signs include: (1) widespread and local pressure pain in the shoulder joint; (2) joint stiffness, such as limited range of shoulder motion (internal and external rotation, forward flexion, abduction, and posterior extension), and increased pain during activity; (3) local muscle contracture, such as contracture of supraspinatus, infraspinatus and deltoid muscles. The goal of surgery is to improve shoulder joint function and relieve clinical symptoms, such as pain, muscle strength, range of motion of the shoulder joint, ability to perform activities of daily living, and local anatomical pattern. Traditional open surgery has largely been abandoned. Arthroscopic capsular release has the advantages of less trauma, fast postoperative recovery, less pain, and good shoulder joint function recovery (4–6), which is gradually more commonly applied clinically. However, during shoulder arthroscopy, extensive and complete capsular release is required in order to achieve good results, so tissue damage, pain, edema and bleeding are unavoidable. Additionally, active and passive functional exercises are required to prevent re-adhesion after surgery, which increases pain, bleeding and swelling to some extent. Patients may be unwilling to perform functional shoulder exercise due to fear of pain, which affects the functional recovery of the shoulder joint. Reducing postoperative pain, bleeding and swelling, accelerating recovery, and maximizing restoration of shoulder function after arthroscopic capsular release are current research hotspots.

Cocktail formula (7, 8) and tranexamic acid (TXA) (9, 10) has been commonly used for intra-articular infusion following hip and knee replacement surgery to relieve pain, reduce bleeding and improve joint function, which have received good clinical results.

For the treatment of frozen shoulder using manipulation (11) and arthroscopic surgery (12), the use of cocktail formula and TXA have received satisfactory clinical results. However, there are few clinical studies on the use of cocktail combined with TXA in arthroscopic capsular release for the treatment of frozen shoulder, clinical efficacy of the combined application of cocktail and TXA needs to be further investigated. Therefore, in the present study, we retrospectively analyzed the clinical data of patients with frozen shoulder who underwent arthroscopic capsular release and received intra-articular infusion of cocktail combined with TXA in Zunyi First People's Hospital from January 2020 to December 2021, observed postoperative pain, intra-articular bleeding, and functional recovery of the shoulder joint in these patients, and aimed to investigate the clinical efficacy of intra-articular infusion of cocktail combined with TXA in the treatment of patients with frozen shoulder after arthroscopic capsular release, so as to provide ideas and new methods for faster recovery in middle-aged and older patients with frozen shoulder after arthroscopic capsular release.

## Materials and methods

### Subjects

A total of 85 middle-aged and older patients with frozen shoulder who underwent arthroscopic capsular release and received intra-articular infusion of cocktail combined with TXA after surgery in Zunyi First People's Hospital from January 2020 to December 2021 were retrospective analyzed. All patients met the diagnostic criteria for frozen shoulder.

The inclusion criteria included: (1) age range from 50 to 70 years; (2) patients who had pain in the shoulder joint with limited ROM (< 80° of forward flexion, <30° of posterior extension, <90° of abduction, <40° of external and internal rotation), Neer score of <70, and Visual Analogue Scale (VAS) score of >6; (3) patients whose symptom did not improved obviously after more than 3 months of conservative treatment (physical therapy) and arthrocentesis; (4) patients' quality of life was affected due to pain and functional limitations, and they had a strong subjective need for treatment; (5) patients who had good general condition and could tolerate the surgical procedures; (6) patients who had good compliance and were able to cooperate with treatment and follow-up.

The exclusion criteria included: (1) patients who had coagulation dysfunction; (2) the presence of infection, tumor, systemic arthritis in the shoulder joint; (3) movement was restricted at the shoulder joint due to sequelae of cerebral infarction; (4) patients who had cervical spondylosis, pain in the shoulder joint and rheumatoid arthritis involving the shoulder joint may be caused by cervical spondylosis; (5) patients who were allergic to anesthetic drugs, TXA and cocktail; (6) patients who had poor compliance, were unable to cooperate with treatment, and lost to follow-up; (7) patients who had shoulder osteoarthritis; (8) patients who had recurrent dislocation of the shoulder, SLAP, Bankart and Hill-sachs lesions; (9) patients whose rotator cuff was of poor quality with massive tears, that may lead a longer postoperative immobilization period.

According to the drugs used for intra-articular infusion after surgery, patients were divided into three groups: a TXA group (*n* = 28), a cocktail group (*n* = 26), and a cocktail + TXA group (*n* = 31).

This study was reviewed and approved by the Ethics Committee of the First People's Hospital of Zunyi City (No. 2019-028), and informed consent was obtained from the patients.

## Methods

### Preoperative preparation

After admission, all patients underwent imaging and relevant preoperative examinations. The extent of adhesions within shoulder joint was identified. The cardiopulmonary function, surgical risk and surgical feasibility in patients were assessed. Each patient completed the visual analog scale (VAS), Neer shoulder assessment scale, and the American Society of Shoulder and Elbow Surgery Shoulder Joint Score (ASES) before surgery to evaluate and record their pain and shoulder joint function.

### Surgical methods

All operations were performed by the same experienced surgeon. All patients received general anesthesia with endotracheal intubation. The surgical table was set into the sitting beach-chair position, and shoulder arthroscopic capsular release was performed. Surgical steps: Step 1: release of the rotator interval composed of long head of the biceps tendon, glenoid lip and the upper edge of the subscapularis, and release of the anterior joint capsule, coracohumeral ligament, superior subscapularis recess, superior and middle glenohumeral ligaments. Particular attention should be paid to release the adhesions at the entrance of the biceps tendon, and protect brachial plexus and vascular bundles during operation. Step 2: release of the anterior inferior joint capsule and the anterior band of the inferior glenohumeral ligament. After this procedure, arthroscopic observation showed that the subscapularis muscle can slide freely during passive external rotation of the glenohumeral joint. If movement was still restricted at the shoulder joint, step 3 was performed. Step 3: release of the posterior joint capsule and the posterior band of the inferior glenohumeral ligament. Care should be taken to protect the underlying axillary nerve (Figure 1A-H).

### Intra-articular infusion of cocktail, TXA and cocktail + TXA

After surgery, a drainage tube was placed in the articular cavity. Drugs were then injected via the drainage tube immediately (Figure 1I). Patients in the TXA group were injected with a mixture of 20 ml of 0.9% sodium chloride injection and 1.0 g of TXA injection. Patients in the cocktail group were injected with 1 ml of compound betamethasone, 5 ml of ropivacaine hydrochloride injection, 5 ml of flurbiprofen axetil injection, 0.5 ml of morphine hydrochloride injection, and 4 ml of 0.9% sodium chloride solution. Patients in the TXA + cocktail group were injected with 4 ml of 0.9% sodium chloride solution, 1.0 g of TXA injection, 1 ml of compound betamethasone, 5 ml of ropivacaine hydrochloride injection, 5 ml of flurbiprofen axetil injection, and 0.5 ml of morphine hydrochloride injection. The drainage tube was clamped immediately after injection was completed.

### Postoperative management

After operation, ice wrap was applied to the surgical area. The drainage tube was opened after 6 h of intra-articular infusion, the amount of drainage was recorded for 24 h, the drainage tube was then removed. After patients were awakened, they were required to performed active and passive functional exercises such as shoulder extension and adduction, abduction and lifting, internal and external rotation. At 1 day after the operation, patients were instructed to use the affected upper limb to perform activities such as climbing the wall, combing the hair, touching the ears, and holding hands behind the back. Each activity was performed 15 times, 3 times per day, for one month continuously.

### Observation indicators

After surgery, drainage volume within 24 h of surgery, postoperative length of hospital stay, postoperative complications, VAS, Neer, ASES scores at 1 day, 1 week, 1 month, and 3 months after surgery were recorded. A goniometer was used to measure and record the maximal ROM of the shoulder joint, including flexion, extension, internal rotation at 90° of elbow flexion, external rotation at 90° of elbow flexion, and abduction before surgery, 1 week, 1 month, and 3 months after surgery.

### Statistical analysis

All statistical analyses were performed using SPSS Statistics version 22.0. Continuous variables (such as VAS, Neer, ASES scores) were presented as mean ± standard deviation (SD). For normal distribution data, independent sample t-test was applied to compare the differences between two groups, and one-way analysis of variance test was applied to compare the difference among multiple groups. *χ*^2^ test was used to compare the difference in rates between groups. P-value <0.05 was considered significant.

## Results

### Clinical characteristics of patients included in the study

A total of 85 patients with frozen shoulder were included in the study, there were 38 males and 47 females. In the TXA group, there were 12 males and 16 females, with an average age of 60.79 ± 4.96 years (range, 54–69 years old). In the cocktail group, there were 11 males and 15 females, with an average age of 61.27 ± 4.45 years (range, 54–69 years old). In the cocktail + TXA group, there were 14 males and 17 females, with an average age of 59.97 ± 5.29 years (range 53–70 years old). There was no significant difference between the three groups with respect to sex, age, body mass index, operation time, preoperative VAS, Neer, and ASES scores (*P* > 0.05, Table 1).

**Table 1:** Comparison of baseline and clinical data of patients among the three groups before surgery.

Baseline and clinical data	TXA group	Cocktail group	Cocktail + TXA group	*χ^2^*/F-value	*P*-value
**Sex**					
Male	12	11	14	0.055	0.973
Female	16	15	17		
Age (year)	60.79 ± 4.96	61.27 ± 4.45	59.97 ± 5.29	0.510	0.602
BMI (kg/m^2^)	22.73 ± 2.06	22.40 ± 1.97	22.71 ± 1.86	0.245	0.783
Operation time (min)	42.04 ± 7.22	41.77 ± 5.76	41.55 ± 5.61	0.045	0.956
VAS score	6.64 ± 0.95	6.58 ± 1.06	6.35 ± 1.03	0.661	0.519
Neer score	34.89 ± 3.65	34.54 ± 3.08	34.45 ± 2.88	0.152	0.859
ASES score	45.04 ± 2.94	44.69 ± 3.79	44.03 ± 3.91	0.601	0.550

TXA, tranexamic acid; VAS, visual analogue scale; ASES, American shoulder and elbow surgery; BMI, body mass index.

### Patient's general condition and imaging findings after surgery

Obvious surgical complications were never observed in the three groups. The average postoperative length of hospital stay was 7.39 ± 1.66) days in the TXA group, 5.46 ± 0.95 days in the cocktail group, 5.16 ± 1.04 days in the cocktail + TXA groups. The postoperative length of hospital stay was significantly shorter in the cocktail + TXA and cocktail groups than that in the TXA group (*F* = 26.519, P = 0.000), while no significant difference was found between between the cocktail + TXA and cocktail groups (P = 0.371, Table 2).

**Table 2:** Comparison of the average postoperative length of hospital stay and 24-h postoperative drainage volume between the three groups (mean ± SD).

Groups	Postoperative length of hospital stay (d)	Drainage volume within 24 h after surgery (ml)
TXA group (*n* = 28)	7.39 ± 1.66	11.57 ± 2.86
Cocktail group (*n* = 26)	5.46 ± 0.95*	19.35 ± 3.55*
TXA + cocktail group (*n* = 31)	5.16 ± 1.04*	10.26 ± 2.49#
*F*-value	26.519	74.803
*P*-value	0.000	0.000

* *P *< 0.05, vs. the TXA group.

# *P *< 0.0, vs. the cocktail group.

TXA, tranexamic acid.

**Figure 2:**
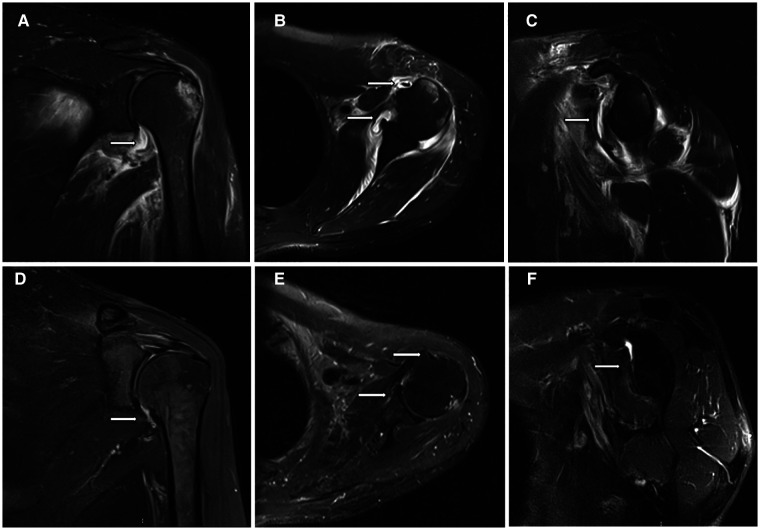
MRI findings of patients with frozen shoulder before and 1 month after arthroscopic capsular release. (**A**) Thickening and edema of the shoulder capsule (white arrow); (**B**) Edema and adhesions surrounding the long head of the biceps tendon and rotator interval (white arrow); (**C**) Thickening of the glenohumeral ligament (white arrows); (**D**) The articular capsule after release (white arrows); (**E**) Long head of the biceps tendon and rotator interval after release (white arrows); (**F**) Removal of the glenohumeral ligament (white arrow).

MRI of the shoulder joint at 1 month after surgery showed that the proliferative, adhesive fibrous and synovial tissues surrounding the shoulder joint had been removed (Figure 2).

### Drainage volume within 24 h after surgery

The postoperative drainage volume was 11.57 ± 2.86 ml in the TXA group, 19.35 ± 3.55 ml in the cocktail group, and 10.26 ± 2.49 ml in the TXA + cocktail group. No significant difference was found between in the TXA and TXA + cocktail group (P = 0.185). Postoperative drainage volume was significantly higher in the cocktail group compared with the TXA + cocktail group (*F* = 74.803, P = 0.000, Table 2).

### Therapeutic efficacy of intra-articular infusion of cocktail combined with TXA following arthroscopic capsular release

Comparison of the VAS scores between the three groups showed that at 1 day and 1 week after surgery, pain was more pronounced in the TXA group, which was significantly relieved in the cocktail and the cocktail + TXA groups, and pain relief was more apparent in the cocktail + TXA groups (*P* < 0.05). Good pain relief was achieved in all three groups at 1 and 3 months after surgery (Table 3).

**Table 3:** Comparison of VAS scores between the three groups before and after surgery (mean ± SD).

Groups	Before surgery	1 day after surgery	1 week after surgery	1 month after surgery	3 months after surgery
TXA group (*n* = 28)	6.64 ± 0.95	5.93 ± 0.81	3.82 ± 0.77	2.07 ± 1.02	1.04 ± 0.69
Cocktail group (*n* = 26)	6.58 ± 1.06	3.73 ± 0.72*	2.96 ± 0.53*	1.23 ± 0.71*	0.88 ± 0.59
TXA + cocktail group (*n* = 31)	6.35 ± 1.03	3.65 ± 0.61*	2.39 ± 0.89*#	1.03 ± 0.69*	0.81 ± 0.48
*F–*value	0.661	92.277	26.911	12.908	1.139
*P–*value	0.519	0.000	0.000	0.000	0.325

* *P *< 0.05, vs. the TXA group.

# *P *< 0.0, vs. the cocktail group.

TXA, tranexamic acid; VAS, visual analogue scale.

Comparison of the Neer scores between the three groups showed that the shoulder joint function was significantly limited in both the three groups before surgery. Shoulder joint function was improved to varying degrees in both the three groups at 1 day after surgery, obvious functional improvement was observed at 1 week after surgery in the three groups, and good level of shoulder joint function was achieved in the cocktail and the cocktail + TXA groups (*P* < 0.05). At 1 month and 3 months after surgery, good or excellent level of shoulder joint function was achieved in all the three groups. Patients in the cocktail + TXA groups obtained excellent functional recovery of the shoulder joint, which was better than the TXA group (*P* < 0.05, Table 4).

**Table 4:** Comparison of Neer scores between the three groups before and after surgery (mean ± SD).

Groups	Before surgery	1 day after surgery	1 week after surgery	1 month after surgery	3 months after surgery
TXA group (*n* = 28)	34.89 ± 3.65	52.39 ± 5.40	72.07 ± 4.67	80.82 ± 3.67	88.04 ± 2.20
Cocktail group (*n* = 26)	34.54 ± 3.08	61.27 ± 4.32*	78.73 ± 3.42*	87.96 ± 2.13*	89.38 ± 2.67
TXA + cocktail group (*n* = 31)	34.45 ± 2.88	70.68 ± 3.02*#	86.71 ± 4.18*#	89.77 ± 2.43*	90.35 ± 2.76*
*F-*value	0.152	132.186	92.530	80.579	6.051
*P-*value	0.859	0.000	0.000	0.000	0.004

* *P *< 0.05, vs. the TXA group.

# *P *< 0.0, vs. the cocktail group.

TXA, tranexamic acid.

Comparison of the ASES scores between the three groups showed that at 1 day after surgery, a certain improvement in the shoulder joint function was achieved in all the three groups. At 1 week after surgery, the shoulder joint function was significantly improved in all the three groups, and the improvement was more apparent in the cocktail + TXA groups (*P* < 0.05). At 1 month after surgery, the cocktail group and the cocktail + TXA groups both showed good functional improvement. At 3 month after surgery, good functional recovery of the shoulder joint was observed in all three groups, the functional recovery of the shoulder joint was better in the cocktail + TXA group than that in the TXA group (*P* < 0.05, Table 5).

**Table 5:** Comparison of ASES scores between the three groups before and after surgery (mean ± SD).

Groups	Before surgery	1 day after surgery	1 week after surgery	1 month after surgery	3 months after surgery
TXA group (*n* = 28)	45.04 ± 2.94	53.43 ± 2.50	62.14 ± 3.91	72.25 ± 4.51	89.04 ± 2.91
Cocktail group (*n* = 26)	44.69 ± 3.79	58.81 ± 3.93*	70.88 ± 2.14*	85.19 ± 2.40*	90.23 ± 2.27
TXA + cocktail group (*n* = 31)	44.03 ± 3.91	59.58 ± 4.62*	78.55 ± 3.19*#	87.03 ± 3.22*	90.90 ± 2.18*
*F-*value	0.601	21.931	194.724	150.981	4.273
*P*-value	0.550	0.000	0.000	0.000	0.017

* *P *< 0.05, vs. the TXA group.

# *P *< 0.0, vs. the cocktail group.

TXA, tranexamic acid; ASES, American Shoulder and Elbow Surgery.

Shoulder ROM was significantly improved in all the three groups at different time points after surgery compared with before surgery, which was gradually increased over time. At 1week after surgery, patients in the cocktail + TXA groups obtained better shoulder ROM (*P* < 0.05). At 1 month after surgery, shoulder ROM was greater in the cocktail and the cocktail + TXA groups than that in the TXA group (*P* < 0.05), and the extension ROM was better in the cocktail + TXA groups than that in the cocktail group (*P* < 0.05). At 3 month after surgery, the ROM was better in the cocktail + TXA groups than that in the TXA group (*P* < 0.05), the ROM for internal rotation, external rotation and abduction was better in the cocktail + TXA group than that in the cocktail group (*P* < 0.05, Table 6).

**Table 6:** Changes in shoulder joint ROM before and after surgery in three groups (mean ± SD, °).

	Flexion	Extension	Internal rotation at 90° of elbow flexion	External rotation at 90° of elbow flexion	Abduction
**Before surgery**					
TXA group	72.93 ± 7.55	22.43 ± 4.11	32.79 ± 4.74	33.07 ± 3.63	83.21 ± 7.08
Cocktail group	73.15 ± 6.19	22.77 ± 2.17	31.85 ± 4.18	32.62 ± 3.18	83.08 ± 6.03
TXA + cocktail group	72.67 ± 6.60	22.20 ± 3.26	32.20 ± 4.17	32.53 ± 4.14	83.27 ± 5.76
**1 week after surgery**					
TXA group	125.36 ± 4.60	32.71 ± 2.64	50.43 ± 4.24	52.86 ± 4.70	126.29 ± 4.68
Cocktail group	132.85 ± 5.79*	34.62 ± 2.69	53.38 ± 3.10	55.77 ± 4.64	131.08 ± 7.25
TXA + cocktail group	137.73 ± 7.00*#	38.67 ± 3.92*#	57.87 ± 4.93*#	58.40 ± 4.85*	135.87 ± 7.51*
**1 month after surgery**					
TXA group	138.07 ± 11.10	42.64 ± 4.05	64.79 ± 6.04	61.93 ± 5.46	140.93 ± 7.25
Cocktail group	150.23 ± 5.86*	44.08 ± 4.52	69.54 ± 4.07*	67.31 ± 4.09*	152.15 ± 7.95*
TXA + cocktail group	154.33 ± 6.41*	47.60 ± 4.63*#	71.93 ± 4.35*	70.67 ± 4.64*	156.93 ± 7.69*
**3 months after surgery**					
TXA group	152.71 ± 8.22	45.57 ± 4.60	68.86 ± 6.19	69.21 ± 3.38	150.93 ± 7.71
Cocktail group	155.54 ± 5.04	46.85 ± 4.28	73.62 ± 3.52*	72.46 ± 4.68*	157.08 ± 5.87*
TXA + cocktail group	157.19 ± 7.71*	50.47 ± 3.52*#	75.13 ± 3.02*	76.27 ± 2.91*#	162.07 ± 6.13*

* *P *< 0.05, vs. the TXA group.

# *P *< 0.0, vs. the cocktail group.

TXA, tranexamic acid; ROM, range of motion.

## Discussion

With the aging of the population in China, the number of patients who suffer from frozen shoulder has gradually increased. Frozen shoulder is the most common cause of shoulder pain, limited shoulder ROM and should stiffness in most middle-aged and older patients, which seriously affects patients' quality of life and their ability to carry out everyday activities (13). Frozen shoulder is a self-limiting condition with an average duration of 2–3 years, but a previous study has shown that 20%–50% of patients still have persistent pain and limited shoulder ROM after 3 years of the onset of frozen shoulder (14), and chronic pain over a long period of time can cause markedly stress on the patient's life and psychological well-being. With the development of society, people's demand for health is increasing, so patients' expectations of being cured of frozen shoulder have also gradually increased. At present, most patients are treated conservatively (15), such as partial closure, release and manipulation under anesthesia (16), and intra-articular injection of drugs (17). Most of patients experience significant temporary relief of symptoms after conservative treatment, with increased shoulder ROM, and improved shoulder function under the action of the drugs. However, with the absorption and metabolism of the drugs, local soft tissue adhesions and original lesions persist, resulting in recurrence and prolonged disease duration.

At present, arthroscopic capsular release is gradually popularized and applied in the treatment of frozen shoulder. Studies have shown that application of arthroscopic capsular release has good safety and efficacy, and can remove the proliferative synovium, adhesive fibrous tissues and damaged tissues in the shoulder joint, and repair damaged tissues (18, 19). The main reasons for pain and limited ROM caused by frozen shoulder are local inflammation and soft tissue adhesion. Therefore, after arthroscopic surgery, patients are required to perform shoulder exercises early to restore the shoulder ROM and improve shoulder function. However, surgical trauma can lead to traumatic pain, and general anti-inflammatory and analgesic drugs are not effective in relieving pain, resulting in poor cooperation with postoperative functional exercises and unsatisfactory surgical results. Some researchers have proposed that functional exercises can be performed after peripheral nerve block, this can obviously reduce postoperative pain, but postoperative nerve block often leads to partial dysfunction of the affected limb, and may have a detrimental effect on the patient's active functional exercise (20). Additionally, postoperative intra-articular bleeding may occur, poor drainage of intra-articular bleeding may lead to haematoma formation in the joint cavity, and aggravation of local inflammation, so as functional exercises progress, pain will gradually increase. And gradual organization of the hematoma will increase the risk of re-adhesion of shoulder joint after surgery.

The cocktail formula (21) has stable and long-lasting anti-inflammatory and analgesic effects, and is widely used in hip and knee arthroplasty to relieve postoperative local inflammation and pain, and enable patients to get out of bed early, restore limb function and achieve faster recovery. TXA (22) has a good postoperative hemostatic effect, and is often used in hip and knee arthroplasty to reduce postoperative intra-articular bleeding and accelerate postoperative recovery. Good clinical outcomes have achieved after using cocktail and TXA in hip and knee arthroplasty, but studies on the application of cocktail and TXA in patients with frozen shoulder after arthroscopic capsular release are rare. Therefore, in the present study, intra-articular infusion of cocktail combined with TXA was performed after arthroscopic capsular release to prevent postoperative pain and the risk of intra-articular bleeding.

In the present study, the surgical procedures were smooth in all the three groups, differences in postoperative bleeding, postoperative pain and postoperative functional recovery were compared between three groups after postoperative intra-articular infusion of cocktail alone, TXA alone, and cocktail plus TXA. There was no significant difference in the postoperative drainage volume between the TXA and the cocktail + TXA groups, but the postoperative drainage volume in the cocktail group was relatively increased, indicating that intra-articular infusion of TXA has a good preventive and control effect on postoperative bleeding, and can reduce postoperative intra-articular bleeding. The degree of postoperative pain in the cocktail and cocktail + TXA groups was significantly relieved, and patients could actively perform shoulder functional exercises with good cooperation, but patients in TXA group had obvious postoperative pain and had to perform functional rehabilitation exercises under intravenous analgesia. The results indicate that the cocktail formula has good anti-inflammatory and analgesic effects, which can relieve acute postoperative pain, accelerate the process of postoperative rehabilitation exercises, and promote the functional improvement of the shoulder. Additionally, the degree of pain was higher in the cocktail group than that in the cocktail + TXA group at 1 week after surgery. This may be related to the slower subsidence of inflammation caused by postoperative intra-articular bleeding in the cocktail group. The comprehensive analysis of shoulder joint function using Neer score, ASES score and shoulder ROM measurement showed that all patients in each group obtained significant functional improvement within 1 week after surgery, and the improvement was more obvious in the cocktail + TXA groups, followed by the cocktail group, indicating that at early time point after surgery, the cocktail combined with TXA can reduce postoperative pain and intra-articular bleeding, and accelerate postoperative functional rehabilitation exercise, so as to promote early postoperative functional recovery of the shoulder joint. At 1 month after surgery, excellent level of the shoulder joint function was achieved in the cocktail + TXA groups, good level of shoulder joint function was achieved in the cocktail group, and significant functional improvement was also found in the TXA group, indicating that with the subsidence of inflammation and the absorption of accumulated blood, the degree of pain gradually decreased, the functional exercises performed by patients gradually increased, and the shoulder joint function gradually improved. At 3 months after surgery, recovery of the shoulder joint function was basically good in all three groups, and the recovery was apparent in the cocktail + TXA groups, indicating that with the strengthening of functional exercises, patients in the three groups both achieved good functional recovery after surgery. The above-mentioned findings suggested that intra-articular infusion of cocktail combined with TXA plays a positive role in functional recovery at all time points after arthroscopic capsular release, especially at the early postoperative period.

Arthroscopic capsular release and intra-articular infusion of cocktail combined with TXA in the treatment of middle-aged and older patients with frozen shoulder have the following advantages: (1) less surgical trauma and faster postoperative recovery; (2) good safety and low incidence of postoperative complications; (3) accelerated postoperative rehabilitation: the combination of cocktail and TXA can reduce postoperative pain, and intra-articular bleeding, allow early functional exercises, thus enabling fast-track postoperative rehabilitation; (4) short length of hospital stay, obvious therapeutic efficacy, and high degree of patient satisfaction.

However, this study also has some limitations. This is a retrospective study, with a relatively short follow-up period. Further studies with larger sample size and long follow-up are needed to investigate the postoperative recurrence rate and the changes in shoulder joint function over time, and to further improve the treatment plan.

In conclusion, our findings suggest that arthroscopic capsular release and postoperative intra-articular infusion of cocktail combined with TXA has good safety and efficacy in the treatment of middle-age and older patients with frozen shoulder, which can reduce postoperative pain and intra-articular bleeding, promote early postoperative functional exercises, and accelerate early postoperative recovery. Intra-articular infusion of cocktail combined with TXA is a safe, effective and fast procedure for the treatment of frozen shoulder, that is worthy of clinical promotion.

## Data Availability

The original contributions presented in the study are included in the article/Supplementary Material, further inquiries can be directed to the corresponding author.
